# On the Process of Absorption in Bone and Tooth Structure

**Published:** 1882-10

**Authors:** Geo. Hilditch Harding


### On the Process of Absorption in Bone and Tooth Structure.

BY GEO. HILDITCH HARDING, L.D.S.

[Read before the Odontological Society of Great Britain.]

Mr. President and Gentlemen :—

The process of absorption is a very interesting one, and especially so to us as dental surgeons, considering what an important part it plays in the various physiological and pathological changes which take place in the dental region, and that it opens up a vast field for future investigations.

The authors who have written upon it are not very numerous, neither do their views entirely coincide as to the manner in which the marvellous changes are effected. Bilroth says: "The cause of the atrophy of bone along the walls of the haversian canals, which takes place in most new formations in the bone, is difficult to explain. The appearances of the connective tissue and muscular substance, as well as of other soft structures when inflammatory new formations occur in them, are less strange; but that hard bony substances should be thus absorbed is truly remarkable."

Now, what is absorption in the hard tissues? I would define it as—

That process by which formed material is reclaimed or retaken up into the system by means of the absorbent cells, known by the the name of "*osteo-clasts.*"

The process may be perfectly healthy and normal, or it may be entirely pathological, resulting from certain peculiar inflammatory conditions.

In illustration of its physiological action I would point out the important part it plays in the development of bone. The alteration in size and in shape of the various adult bones, as compared with those of the fœtus, is accomplished through the influence of absorption and deposition. The lower jaw is a good example of this: in the fœtus the angle is very obtuse, whereas in the adult it assumes almost a right angle, and in old age we again see it assuming the obtuse angle, this great change in shape being effected by the combination of those two forces.

Now, with regard to the increase in size of the adult bone compared with the fœtal bone, this change is brought about in a similar manner; the external contour is increased by deposition taking place beneath the periosteum, and also beneath the articular cartilages, whilst at the same time absorption is taking place in the internal of the bone; the interior layers are gradually being changed from the compact tissue into the cancellous, and so the medullary canal is increased in calibre, and as deposition is more active during the period of development than that of absorption, the walls of the medullary canals are increased in thickness.

Proceeding with this subject from its physiological aspect, I will examine briefly the great changes which take place in the dental alveoli.

During the formation of the teeth we find the formative process very active, and the bone is being built up around them until they are enclosed in bony cells or crypts; and if we examine them again at a latter period, we find that the process of absorption has set in, by which the walls of these crypts are removed to allow of the eruption of the contained teeth.

The crypts which contain the incisors have their anterior walls removed, so as to expose the labial surfaces of the crowns of those teeth; whereas in the crypts of the molars that portion of the wall covering the points of the cusps is the first to be removed. After the teeth have erupted, the formative process again sets in, and the bone is built up around the erupting teeth, so that when they are fully erupted their fangs are encased in bony sockets. The deciduous set, together with their alveoli, are removed by the advent of this absorbent process. In the incisors we find absorption as a rule taking place on the posterior surfaces of their fangs, and commencing near their apices; and in the molars absorption takes place in the upper jaw on the internal surfaces of the palatine and buccal fangs, which approximate the erupting bicuspids, and in the lower jaw the anterior surface of the posterior fang, and the posterior surface of the anterior fang, is the first to be removed. I will refer to this subject further on in my paper. In old age again, when these useful organs are too apt to throw off their mortal coils, or become the victims of the forceps or the elevator, their former habitations are removed by the absorbent process, so that in the edentulous jaw there eventually remains little but the dense compact tissue of the body of the bone. Having glanced very superficially, at the prominent part this process plays in a few of the physiological changes which occur in the hard times, it will be well to consider its connection with certain pathological conditions and actions; but before doing so, I will describe the histologica appearances presented, and some of the various theories advanced as to the method by which the lime salts are removed.

If an absorbed surface be examined by a moderately powerful magnifying-glass, it will present a rough, uneven, jagged, or as one author aptly describes it, "a gnawed appearance," and the surface will be covered with pits or excavations. If a section be cut through an absorbed surface at right angles to it, the edge of the section which has been acted upon will present certain peculiarities. It will appear to be made up of a series of arcs of circles, as though round pieces had been punched out of the edge; the spaces between these indentations being occupied by the sharp projections formed by the edges of the adjoining circular excavations.

I believe that Howship was the first to notice and investigate the nature of these excavations, and consequently they have received the now familiar name of *Howship's Lacunæ.* There is, in contact with a surface where absorption is taking place, a new formation which is richly supplied with cells; this is the absorbing organ. The cells are of the compound order.

In connection with the absorption of the temporary teeth, the new formation which is there present, and is identical with that found elsewhere in many respects, has been called by Tomes the "Absorbent Papilla," and it would be difficult to give a better description of it than the one given by that author; he says: "The surface is made up of peculiar multiform cells, each one being composed of several smaller cells, the number varying from two or three to as many as fourteen or fifteen. The form is variable, but egg-shaped or spherical figures are found to prevail, although some few deviate from these forms and offer a very strong resemblance to those cells described by Koelliker as myeloid cells." If a section be made carefully from a fresh specimen, with the aid of some decalcifying agent, it will be seen these pits or excavations are occupied by cells. Sometimes it will be seen that one excavation will contain one cell, at others two or three.*

* Rustizky found that if he applied vermilion to the living tissue where these cells were present, the cells took up the granules into their substance.

Volkmann describes certain changes, to which he has applied the name of *canaliculization,* and which takes place in the bony tissue previous to the advant of the absorbent process. He says that in examining sections at this period he has observed numerous canals ramifying through the tissue, which canals make numerous communications forming a very complete anastomosis. The outlines of these canals present numerous projections, which very much resemble the margins of bone lacunæ; and as these canals run in the same direction as the bone lacunæ and occupy the position former lacunæ which have been destroyed, he considers that they are due to the increased flow of blood in the part, consequent upon inflammatory conditions existing; and that the canalicule of the bone cells by which the circulation in the bone is ordinarily maintained, become enlarged in order to meet the increased flow of blood which takes place. Volkmann thought that these canals were occupied by capillary vessels. Rindfleisch has also observed this canaliculization, but does not support this latter view. He says:

"I have never succeeded in injecting them from the blood-vessels, or in finding the characteristic elements of a capillary tube, such as a nucleated membrane, in their interior. Judging from the way they refract light, the contents of the canals must be identical with that of the bone lacunæ; hence I can only regard them as a further development of the nutrient apparatus provided by the system of anastomosing lacunæ."

Bilroth says: "At present it is not known how the lime salts are dissolved in this process; I think the new formation in the bone develops lactic acid, which changes the carbonate and phosphate of lime into soluble lactate of lime, and which is taken up and removed by the vessels."

Rindfleisch is of opinion that a solution of the intercellular substance takes place in the fluid. It has been a source of some controversy as to the manner in which *Howship's lacunæ* are formed. Virchow and others were of opinion that each of these excavations corresponded to the nutrient territory of a bone-corpuscle. Bilroth says he is convinced that the bone-corpuscles have as little to do with this process as the fixed connective-tissue cells.

I think that the fact of our finding these *Howship's lacunæ* in dental tissues which possess no bone-corpuscles (viz., dentine and enamel), would tend to disprove this statement altogether.

Passing on from its physiological characteristics we may consider this process from its pathological aspect.

Take first of all a case of *osteoplastic ostitis*, as an example of which I will consider a case of fracture, seeing that the inflammation set up is of that character. As a result of the injury, the tissues in the immediate neighborhood become inflamed, a cellular exudation takes place in the medulla, on the surface of the bone and in the haversian canals, and capillary loops form. The cells appear to collect upon the walls of the haversian canals, and the flow of blood is greatly increased. The haversian canals become enlarged by the gradual absorption of their walls; the cell-proliferation goes on as absorption makes room for it, and so the neoplasia thus formed in the two pieces of bone eventually meet and unite the fractured surfaces. If a section be made of a bone at this stage, *Howship's lacunæ* will be plainly visible. Now when the required amount of neoplasia has been thrown out, ossification sets in.

This new formation may be either transformed into cartilage or it may ossify directly, and the new bone be deposited on the absorbed surfaces of the haversian canals, making the canals of the two fractured surfaces continuous; at the same time the neoplasia in the medulla and beneath the periosteum on the surface of the bone is undergoing ossification, forming the external and internal callus. If a section be made at this stage, the line of absorption in the haversian canals can be discerned with the new bone deposited upon its surface. After a fractured bone has united, great changes take place in its shape and structure. There is great difference in the amount of callus which is formed in the various bones.

In the flat bones there is not much callus thrown out, and in those of the skull there is scarcely any formed at all; but the surfaces appear to be glued together, and the union which takes place in them is chiefly affected by the callus, which is formed in the Haversian canals at the expense of their walls, in the manner previously described. The amount of callus formed in some of the long bones is very considerable. The structure of newly-formed callus is of a very porous nature; this eventually becomes consolidated, and the superfluous callus is removed by absorption.

In long bones, where union has taken place whilst much displacement of the fragments existed, the masses of callus thrown out are so enormous that one can hardly imagine such development taking place, were there not specimens to illustrate it.

Now, what is still more remarkable is, that in some of these cases, notwithstanding the gigantic masses of callus and great displacement of the fragments which exist, absorption and deposition so work together, hand-in-hand, sometimes for years, that eventually the bone becomes remodelled, the superfluous callus being removed where required, so that the original shape is nearly restored, and a new medullary canal tunnelled out in the place of the one obliterated by the fracture.

There are cases on record, or one would be unabled to realize that such marvellous changes could be effected.

Bilroth states that where a fracture takes place in a spongy bone the bony cells become filled with bony substance, but that this is eventually removed by absorption.

In "caries," which is in reality a chronic inflammation of the connective tissue of the bone, accompanied by a wasting of the compact osseous tissue of the bone, we again find the granulating tissue being developed and absorption taking place.

The individual trabeculæ of bone become absorbed, and so cut off and removed. If a small flake of bone be removed from a carious spot, and be examined under the microscope, *Howship's lacunæ* will be seen on its edge; and should a section also be made from the bone in the immediate neighborhood of a carious spot, and the lime salts removed by chromic acid, it will appear to be eaten away, and the carious edges will present the characteristic excavations met with on an absorbed surface, and fitting closely into these irregularities will be seen the new granulation-tissue.

In *necrosis* the portion of dead bone is separated from the living by the assistance of this process. Inflammatory action takes place in the adjacent living bone, granulation-tissue is thrown out, which removes the lime salts from the line of demarcation, and from the walls of the haversian canals, which latter become consequently enlarged.

After the necrosed portion is thrown off, ossification sets in, and the haversian canals form fresh communications, and so the circulation is maintained again, which was of necessity previously interrupted.

On microscopical examination it will be seen that the dead portion of bone which has come away has undergone absorption, as well as the living surface from which it has been detached.

There is only one more morbid process in bone to which I will allude, and that is the peculiar disease known as *mollities ossium.* Here we find the bone not diminished in size, nor altered in shape, but exceedingly brittle and porous in structure; it is easily cut or bent.

Rindfleisch says:—

"If we break off a minute trabecula from the cancellous texture of a bone affected with this disease, and, after soaking it in carmine, examine it under a magnifying power of 300 diameters, we find a highly characteristic set of appearances. The trabecula is seen to consist of two very different substances; it exhibits two very distinct zones—an outer one lying next the medullary spaces and the haversian canals, and an inner one, which forms the axis of the trabeculæ.

The inner zone consists of perfectly normal bone-tissue. The corpuscles with their countless anastomosing prolongations, the highly refracting colorless basis substance, are unaltered.

The outer layer, on the other hand, exhibits a finely striated basis substance deeply stained with carmine, in which only a few scattered streaks of shadow indicate the former position of the bone-corpuscles, and of their processes not a vestige remains." The line of demarcation between these two zones is very decided, and exhibits the same semicircular excavations which are met with in other inflammatory processess which occur in bone-substance.

Green, notices one or two rather interesting facts in connection with this disease. He states that the medullary tissue contains fat, that lactic acid is found in the bone and also in the urine, and that lime salts are eliminated by the kidneys.

I should like to say more in connection with this subject in its relation to bone, but in order not to make my paper too lengthy I will pass on to the various dental tissues. It will be seen that here, as in bone, it may assume entirely a physiological action, or the process may be the result of inflammatory conditions. As an instance of the former, the shedding of the temporary teeth forms a good illustration.

Some hold the opinion that the deciduous tooth is removed by pressure exerted by the coming permanent tooth; but there is such abundant proof of the inaccuracy of this statement, that I do not think it can be accepted. One of the most convincing arguments against it is one advanced by Charles Tomes; it is this: that as the fang of the deciduous tooth becomes absorbed so the socket closses up behind it by the deposition of new bone; so that if the socket of a deciduous tooth, about to be shed, be examined, it will be found that a plate of bone exists between it and the coming permanent tooth, so that no pressure could, under those circumstances, be exerted.

Spence Bate, in a communication he once made to this society, advanced the theory that in the removal of the deciduous set the enamel-organ of the permanent tooth performed the office of an absorbent organ; but this is again incorrect, for at this period the enamel-organ of the permanent tooth has undergone calcification.

I think it has been pretty clearly recognized now, that for the removal of a deciduous tooth a proper absorbing organ is formed, called the *absorbent papilla*, the structure of which was described in the early part of this paper. This organ is accurately adapted to the absorbing surface, and on the removal of a temporary tooth we often find a portion of this organ torn away and adhering to the absorbed surface.

The appearances presented in the dental tissues are very similar to those seen in bone; but there are several differences which will require some consideration.

Of the three dental tissues, only one can be replaced after its removal by absorption, by a similar tissue, viz., the cementum, or crusta petrosa. The enamel and dentine, when once removed by this process, can never be replaced by the same tissue, although they may be, and often are, replaced by cementum.

Microscopic examination clearly illustrates this result; a favorable specimen will sometimes show the *granular layer of Tomes* broken by the action of absorption, and the gap entirely filled by cementum; and the former line of absorption separating the two tissues with Howship's lacunæ, plainly discernible, will be readily recognized. * * * *

The process of absorption in temporary teeth takes place alternately with that of deposition. When bone becomes absorbed, new bone is often deposited on the absorbed surface, leaving only a light tracing to indicate where the former line of absorption once existed. The same is true of cementum, but not of the other two tissues, as has already been illustrated; in these, when once the process of deposition sets in, entirely a different tissue is deposited, forming a definite division.

The other characteristics which are presented microscopically in tooth structure very much resemble those which have been described in bony tissue.

In absorption of temporary teeth all three tissues may be attacked, and in the following order:—

1st. The cementum.
2d. The dentine.
3d. The enamel.

We frequently find in practice that only a portion of the crown of a deciduous tooth remains, the greater part of the tooth having become absorbed.

Tomes is of opinion that in the later stages of absorption of the deciduous teeth the pulp takes upon itself an absorbing action, and so completes the work of the papilla.

Absorption in the permanent set must be looked upon as a morbid process. It generally arises as the result of chronic inflammatory conditions existing in the dental periosteum, or the result of irritation set up by impacted permanent teeth. With regard to that arising from chronic periostitis, it occurs usually, I think, in advanced life, and in those teeth the pulps of which have lost their vitality, the process of absorption being often succeeded by exostosis. Stumps which have been pivoted nearly always eventually undergo absorption, and some instances have occurred where the pin of the pivot-tooth has been exposed in this manner. The fangs of permanent teeth which have undergone absorption *nearly always* present that transparent appearance when held up to the light to which Salter has applied the name of "*the horny fang.*' In sections made from such teeth the structure is extremely indistinct, and the characteristics of absorption cannot be traced. The occurrence of cases where the fangs of a permanent tooth have been removed whilst the crowns have remained perfectly healthy is not nearly so frequent. The fang may be almost entirely removed, leaving little but the crown of the tooth remaining, and not given rise to much pain or discomfort until the tooth becomes loose, and eventually falls out; but sometimes, long before it has reached this advanced stage, considerable pain and irritation is set up, which in many cases necessitates its extraction. On examination, it will be frequently found that absorption has been going on in a very irregular manner, leaving the apex of the fang extremely rough, and in many instances it will be found that quite a sharp spicula of dentine projects, which has received the common name of the "*needle point.*" In absorption of permanent teeth in this manner, the dentine and cementum, I believe, are the only tissues attacked; at least, I have never met with a case in which the enamel has been implicated, the tooth being lost before that tissue was reached. In these cases I do not know of any treatment that will arrest the progress of the disease. With regard to absorption of permanent teeth caused by impaction, I believe those most frequently affected in this manner are the twelve-year-old molars, by irritation set up by an impacted wisdom tooth pressing upon its posterior surface. Serious mischief is often set up in this manner, and not unfrequently the pulp-cavity is laid bare, giving rise to the most acute pain and various other complications, which are sometimes very extensive; and under such circumstances I would advocate the extraction of the twelve-year-old molar, but should affairs not have assumed so grave an aspect, the removal of the wisdom tooth would in all probability be the proper course to adopt.

As disturbance may be set up by the occurrence of this process in the second set, so the same may result in the deciduous set from the arrest of it. In the front incisors it is not an unfrequent occurrence for the apices of necrosed, non-absorbed fangs to be exposed by the absorption of the gum over them, and for them to protrude, causing very painful and sometimes extensive ulcers on the inner surface of the upper lip.

Alveolar abscess and gumboil are also very frequently met with in connection with necrosed temporary teeth, which often assume a severe character, and are accompanied with great swelling, etc., etc.

Ragged temporary teeth and stumps that have not been removed by absorption are apt to cause severe ulcers on the tongue.

Lastily, the non-absorption of temporary teeth causes great irregularity in the permanent set. The teeth which I think most frequently assume the persistent phase are the temporary molars and canines; and one can readily see what crowding the retention of the temporary molar would cause, even though its successor did not make its appearance, from the fact of its being so much larger than the pre-molar. Sometimes the persistent temporary tooth is retained for many years, to the exclusion of its successor, and does good service during that period. I have met with instances where a certain temporary tooth has remained persistent in each member of a family. Now the question arises whether, if there is not much crowding, it is the better practice to allow these teeth to remain or to extract them? I think that in many instances, if they be extracted, the gap may remain for a considerable period unoccupied; and should the bicuspid happen to be buried deeply in the jaw, there is some probability of the crowns of the adjoining teeth approximating, and so sufficient space not being maintained for it.

How is it that the absorbing process is sometimes arrested?

Some have advanced the theory that dead tissue cannot undergo absorption; but I do not think that will hold good, seeing that, in Dieffenbach's operation in pseudarthrosis, the ivory pegs are removed in this manner. In this operation the ends of the bone are perforated, and ivory pegs are driven in with a hammer; new bone forms around them. When these pegs are extracted, they will be found to be very rough at the points where they were in contact with the bone; if these pegs be not removed, they become entirely absorbed. On microscopical examination, this ivory presents the typical appearances of absorption, Another proof of the fact that dead tissue can be absorbed is, that the dead bone which is exfoliated in *necrosis* and *caries* also presents the same appearances.

Then, how is it that we find necrosed teeth do not become absorbed?

One would think that it is not so much due to the loss of vitality in the tooth, or to any chemical change taking place in its substance, as to some alteration in the character and functions of the absorbing organ.

